# Ether-Functionalized Polybenzimidazole Composite Separators for Enhanced Performance and Sustainable Lithium-Ion Batteries

**DOI:** 10.3390/ma19122469

**Published:** 2026-06-09

**Authors:** Zhike Li, Wenxuan Li, Hongmin Zhang, Shiman Zhang, Caihong Xue

**Affiliations:** Qinghai Provincial Key Laboratory of New Light Alloys, School of Mechanical Engineering, Qinghai University, Xining 810016, China; m17638373625@163.com (Z.L.); l1572686083@163.com (W.L.); hmz010920@163.com (H.Z.); zhangshiman@qhu.edu.cn (S.Z.)

**Keywords:** lithium-ion battery, separator, electrospinning, polybenzimidazole, electrochemical properties

## Abstract

Polybenzimidazole (PBI) is a promising separator for lithium-ion batteries (LIBs) owing to its excellent thermal/chemical stability and mechanical strength, but its application is limited by poor solubility and processability. Herein, a novel ether-functionalized PBI was synthesized, and three-layer composite separators (PBIPHPE) were fabricated by electrospinning PBI/PVDF-HFP blends onto polyethylene (PE) substrate. The PBIPHPE separator exhibits high porosity (73.1%), superior electrolyte uptake (211.2%), and excellent ionic conductivity (1.125 mS/cm), with no dimensional change after thermal treatment at 150 °C for 0.5 h. Lithium-ion batteries assembled with PBIPHPE deliver an initial specific capacity of 157.7 mAh/g, retain 86.0% capacity after 400 cycles at 2 C, and show only 15.7% capacity decay from 0.2 C to 5 C. Molecular dynamics simulations of the composite separator–electrolyte system were performed to reveal Li^+^ transport behaviors. The results confirm that ether-functionalized PBIPHPEs enhance Li^+^ transport and cycling stability, providing a promising route for high-performance separators.

## 1. Introduction

Owing to their superior cycling stability, high energy density, and environmental compatibility, LIBs have been extensively utilized in portable electronic devices, including smartphones and laptop computers [1,2,3]. Nevertheless, the rapid expansion of electric vehicles and related emerging technologies has subjected the safety characteristics of LIBs to unprecedented challenges [4]. As a critical component that physically separates the cathode and anode, the separator not only enables efficient Li^+^ transport while preventing internal short circuits but also plays an indispensable role in safeguarding the operational safety of LIBs [5,6]. Owing to excellent mechanical strength and low manufacturing cost of commercial separators, specifically PE and polypropylene (PP), these polyolefin-based separators remain the most extensively utilized separator products in the current market [7,8]. However, commercial microporous polyolefin-based separators exhibit several non-negligible shortcomings. In particular, their poor affinity for liquid electrolytes leads to limited electrolyte absorption and an increase in interfacial impedance. Consequently, Li^+^ transport through the separator is hindered, which adversely affects the rate capability and cycling performance of the resulting LIBs [9,10,11]. Furthermore, commercial polyolefin-based separators exhibit inadequate thermal stability and are prone to severe thermal shrinkage at temperatures ranging from 120 to 150 °C. Such dimensional instability can lead to internal short circuits, thereby posing serious safety risks in LIBs [12,13,14]. Consequently, LIBs incorporating conventional commercial separators are ill-suited for safe operation under extreme high-temperature conditions. To address the escalating demand for high-performance LIBs in applications such as electric vehicles, it is imperative to enhance the electrolyte wettability of commercial separators, enhancing Li^+^ transport across the electrode–separator interface.

To overcome these limitations, ceramic-coated separators and electrospun nanofiber separators have been intensively investigated. Ceramic nanoparticles such as boehmite, silicon dioxide (SiO_2_), zirconium dioxide (ZrO_2_), and titanium dioxide (TiO_2_) can be coated onto polyolefin separators to improve thermal resistance and electrolyte affinity [15,16,17,18]. However, the ceramic layer inevitably increases the overall thickness, partially blocks the pores, and shows weak adhesion to the substrate, leading to compromised energy density and detachment during long-term cycling [19,20]. Electrospinning is a versatile technique that produces highly porous nanofiber separators with interconnected pore networks, facilitating electrolyte uptake and Li^+^ transport [21,22,23,24,25]. Currently, the most extensively investigated polymers for electrospinning applications include poly(vinylidene fluoride) (PVDF), PVDF-HFP, polyacrylonitrile (PAN), and poly(methyl methacrylate) (PMMA) [26,27,28,29]. PVDF-HFP is particularly attractive because of its high dielectric constant, low surface energy, and robust C–F bonds that ensure chemical stability [30,31]. Nevertheless, electrospun PVDF-HFP separators still lack sufficient mechanical robustness and thermal stability, undermining battery safety [32,33]. To address these limitations inherent to electrospun PVDF-HFP separators, considerable research efforts have focused on strategies including composite modification and blending with other polymers.

PBI refers to a class of rigid aromatic heterocyclic polymers characterized by benzimidazole repeating units along the polymer backbone. Owing to its exceptional thermal stability, chemical inertness, and superior mechanical strength [34], PBI has been successfully employed in the fabrication of LIB separators. Despite these advantages, conventional PBI suffers from low molecular weight, poor solubility, and processing difficulty, which restrict its direct application. Strikingly, the rational design of novel PBI molecular architectures to simultaneously achieve excellent thermal stability and high electrochemical performance in LIB separators has rarely been investigated—representing a critical research gap. Current approaches to fabricate PBI separators mainly rely on physical processing strategies such as induced phase separation [35], nanophase separation [36], phase inversion [37], and the synthesis of hyperbranched polybenzimidazole (HBPBI) [38]. Although effective to some extent, these methods do not engineer the intrinsic molecular structure of PBI to optimize separator properties. The absence of molecularly designed PBI separators thus severely limits the development of next-generation LIBs that simultaneously deliver superior safety and electrochemical performance.

In this work, a novel synthetic strategy was devised to incorporate ether groups into the repeating units of the PBI main chain by inserting ether linkages between the benzene rings of the tetra-amine monomers. This structural modification effectively modulates the spatial conformation and packing arrangement of the polymer chains, thereby substantially enhancing the flexibility and processability of the resulting PBI. Subsequently, PBIs with varied molecular architectures were dissolved together with PVDF-HFP in N,N-dimethylacetamide (DMAC) to form homogeneous electrospinning solutions, from which nanofiber composite layers were deposited onto both surfaces of a PE separator via electrospinning. Benefiting from the intrinsically outstanding thermal stability of PBI, the composite separator exhibits markedly improved thermal resilience when PBI serves as the structural framework of the nanofiber layer. Concurrently, the proton-donating amine groups (–NH–) are capable of forming hydrogen bonds with the anions of the lithium salt. This interaction effectively immobilizes the anions, enhancing the composite separator’s electrolyte wettability and accelerating Li^+^ transport kinetics. Molecular dynamics simulations were conducted on the composite separator–solvent system to elucidate the Li^+^ migration behavior.

## 2. Materials and Methods

### 2.1. Materials

4-Chloro-1,3-dinitrobenzene (C_6_H_3_ClN_2_O_4_, 90%) was purchased from Shanghai Macklin Biochemical Technology Co., Ltd. (Shanghai, China). Hydroquinone (C_6_H_6_O_2_, 99%) was obtained from Shanghai Macklin Biochemical Technology Co., Ltd. Hydrazine hydrate (N_2_H_4_·H_2_O, 80%) was supplied by Tianjin Obokai Chemical Co., Ltd. (Tianjin, China). Isophthalic acid (C_8_H_6_O_4_, 98%) was purchased from Shanghai Macklin Biochemical Technology Co., Ltd. Acetone (CH_3_COCH_3_, AR) was acquired from Sinopharm Chemical Reagent Co., Ltd. (Shanghai, China). N,N-Dimethylformamide (C_3_H_7_NO, 99.5%) was obtained from Sinopharm Chemical Reagent Co., Ltd. N-Methylpyrrolidone (NMP, electronic grade, 99.9%) was purchased from Shanghai Aladdin Biochemical Technology Co., Ltd. (Shanghai, China). Lithium iron phosphate (LiFePO_4_, battery grade) was supplied by Qinghai Taifeng Advanced Lithium Energy Technology Co., Ltd. (Xining, China). Polyvinylidene fluoride (PVDF, HSV900) was obtained from Arkema, France. (Puteaux, France). Acetylene black was purchased from Shenzhen Tianhe Technology Co., Ltd. (Shenzhen, China). PE separator was supplied by Qinghai Beijie New Material Technology Co., Ltd. (Xining, China). Liquid electrolyte (1 M LiPF_6_ dissolved in ethylene carbonate/dimethyl carbonate/ethyl methyl carbonate, EC/DMC/EMC = 1:1:1, vol%) was purchased from Dongguan Kelude Innovation Technology Co., Ltd. (Dongguan, China). Acetylene black was obtained from Shenzhen Tianhe Technology Co., Ltd. Platinum-on-carbon (Pt/C) catalyst was purchased from Shanghai Macklin Biochemical Technology Co., Ltd. Polyphosphoric acid (H_n+2_P_n_O_3n+1_, 85%) was purchased from Shanghai Macklin Biochemical Technology Co., Ltd. Poly (vinylidene fluoride-co-hexafluoropropylene) ((-CH_2_CF_2_-)_n_[-CF_2_CF(CF_3_)-]_m_, average M_w_~400,000) was purchased from Shanghai Aladdin Biochemical Technology Co., Ltd.

### 2.2. Synthesis of PBIs

In a 250 mL three-necked flask equipped with a mechanical stirrer and a nitrogen inlet, 14.2 g of 4-chloro-1,3-dinitrobenzene, 3.3 g of hydroquinone, 9.0 g of potassium carbonate, and 80 mL of N,N-dimethylformamide (DMF) were combined. The reaction mixture was heated to 140 °C and maintained under a nitrogen atmosphere with continuous mechanical stirring for 24 h. Upon completion, the reaction mixture was poured into a large excess of distilled water to induce precipitation. The resulting precipitate was collected and washed thoroughly with distilled water (three times). The obtained solid was dried in a vacuum oven (Shanghai Yiheng Scientific Instrument Co., Ltd., Shanghai, China) at 40 °C for 24 h to yield the intermediate 1,4-bis(3,4-dinitrophenoxy) benzene (DNPB).

**Figure 1:**
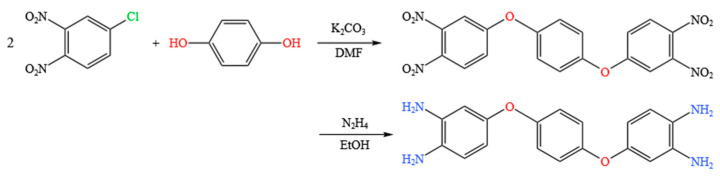
Synthesis of 1,4-bis (3,4-diaminophenoxy) benzene.

In a 500 mL three-necked flask, 8.84 g of DNPB, 1.6 g of platinum-on-carbon (Pt/C) catalyst, and 200 mL of ethanol were charged. Under a nitrogen atmosphere, 60 mL of hydrazine hydrate was added dropwise with caution. The mixture was then heated to 80 °C and magnetically stirred for 24 h. After the reaction was complete, the Pt/C catalyst was removed by filtration, and the filtrate was poured into distilled water. The resulting precipitate was isolated via high-speed centrifugation and subsequently dried in a vacuum oven at 40 °C for 24 h to afford the final product 1,4-bis(3,4-diaminophenoxy) benzene (DAPB), the synthetic route is illustrated in Figure 1.

**Figure 2:**
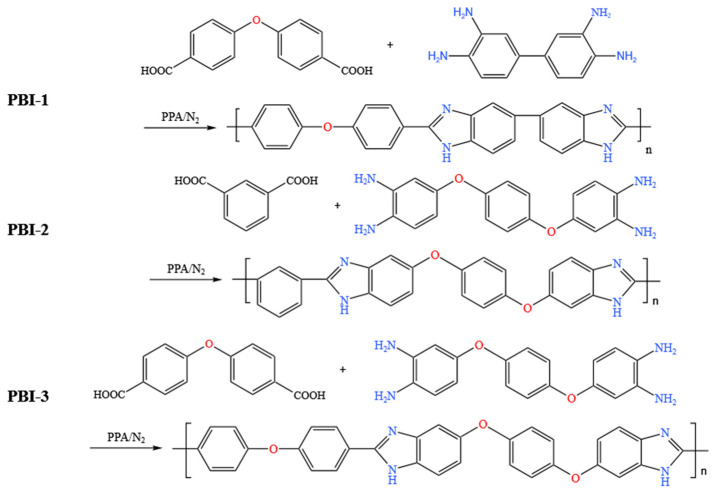
Synthesis of PBI with different structures.

First, 100 g of polyphosphoric acid (PPA) was charged into a three-necked flask and heated to 120 °C under a continuous nitrogen purge. The temperature was maintained for 1 h to eliminate entrapped bubbles within the PPA. Subsequently, 9.3 mmol of dicarboxylic acid and 9.3 mmol of tetra-amine monomer were introduced into the flask. The temperature was gradually raised to 150 °C and held for 3 h to ensure complete dissolution of the monomers. The mixture was then heated to 170 °C and maintained for 2 h to initiate the preliminary polycondensation reaction, followed by further heating to 190 °C. The polymerization was allowed to proceed until a marked increase in the viscosity of the reaction medium indicated the completion of the reaction, the synthetic route is illustrated in Figure 2.

The resulting viscous solution was carefully poured into distilled water under continuous stirring to precipitate the polymer. The collected precipitate was washed with distilled water (2–3 times) and subsequently immersed in an excess of a 10 wt% aqueous sodium bicarbonate solution to neutralize and remove residual PPA. The immersion was maintained until no further gas evolution was observed, indicating complete neutralization of the residual polyphosphoric acid. Subsequently, the precipitate was washed repeatedly with distilled water until the washings became neutral. Following neutralization, the product was dried overnight in a vacuum oven at 120 °C to afford the target polybenzimidazoles, designated as PBI-1, PBI-2, and PBI-3, respectively.

### 2.3. Preparation of Composite Separators

A mixed solvent of DMF and acetone with a mass ratio of 7:3 was prepared, and 12 wt% PVDF-HFP powder was added. The mixture was magnetically stirred for 8–12 h to achieve complete dissolution, affording a homogeneous electrospinning solution. A polyethylene PE separator (the thickness is 8 μm) was fixed onto the rotating collector of an electrospinning apparatus (Beijing Yongkang Leye Co., Ltd., Beijing, China). An appropriate volume of the spinning solution was loaded into a syringe and electrospun onto one side of the PE separator. Upon completion, the resulting separator was transferred to a vacuum oven and dried at 60 °C for 12 h. The dried separator was then removed, and the electrospinning procedure was repeated on the opposite side to yield a PVDF-HFP/PE/PVDF-HFP three-layer composite separator, the thickness of the separator is 27 μm (hereafter designated as the PHPE separator) (the electrospinning voltage was 20 kV, the feeding rate was 1 mL/h, the tip-to-collector distance was 15 cm, and the rotation speed of the collector was 100 r/min).

**Figure 3:**
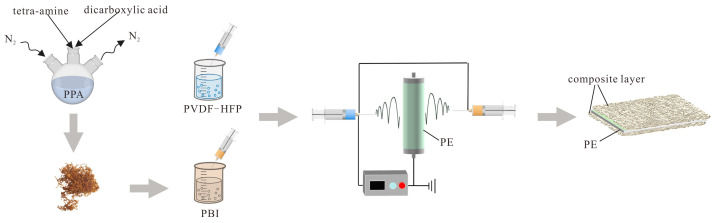
Dual-spinneret electrospinning for the fabrication of composite separators.

Different from the preparation of the aforementioned PHPE separators, the solvent used for the PBI spinning solution was a mixture of DMAC and acetone in a mass ratio of 7:3. The mixture was magnetically stirred at 60 °C for 8–12 h to ensure complete dissolution. Subsequently, a certain volume of the PVDF-HFP spinning solution and the PBI spinning solution were each drawn into two syringes, and a dual-needle electrospinning setup was employed to fabricate PBI@PVDF-HFP/PE/PBI@PVDF-HFP three-layer composite separators, the thickness of the separator is 27 μm (hereafter designated as the 1PBIPHPE, 2PBIPHPE, and 3PBIPHPE separator); a schematic diagram is presented in Figure 3.

### 2.4. Characterization and Physical Performance Measurements

Fourier transform infrared (FTIR) spectroscopy was performed on a Nicolet 6700 spectrometer (Thermo Fisher, Waltham, MA, USA). Samples were intimately ground with KBr at a mass ratio of 1:100, and spectra were recorded over the wavenumber range of 400–4000 cm^−1^ with 64 scans.

^1^H nuclear magnetic resonance (^1^H NMR) spectra were acquired on an Avance III 500 MHz superconducting NMR spectrometer (Bruker, Bremen, Germany) using deuterated dimethyl sulfoxide (DMSO-d_6_) as the solvent. Spectra were collected with 16 scans at a resonance frequency of 600 MHz, covering a chemical shift range of 0–14 ppm.

Morphological observations were carried out using a JSM-7900F field-emission scanning electron microscope (FE-SEM, JEOL, Tokyo, Japan). Prior to imaging, all samples were sputter-coated with gold for 80 s. Micrographs were obtained at an accelerating voltage of 15 kV under backscattered electron mode (LA0 level).

The solubility of the synthesized PBIs was evaluated by introducing the polymers at loadings of 5 wt% and 10 wt% into separate test tubes containing NMP, DMF, DMAC, and DMSO, respectively, at 25 °C. The dissolution behavior of each sample was visually monitored to assess its solubility.

The separators were cut into circular disks with a diameter of 19 mm and subsequently immersed in n-butanol for 2 h to achieve complete saturation. Upon removal, residual n-butanol on the separator surface was gently blotted away with filter paper. The porosity of the separator was then calculated according to Equation (1):

(1) $$\mathrm{Porosity}\;\left(\%\right)\;=\;\frac{m_{w}\;-{\;m}_{d}}{\rho V}\;\times \;100\%$$

where m_w_ is the wet mass of the separator after immersion in n-butanol for 2 h (g), m_d_ is the dry mass of the separator (g), ρ denotes the density of n-butanol (g/cm^3^), and V represents the geometric volume of the separator (cm^3^).

The separator was cut into circular disks with a diameter of 19 mm and immersed in the liquid electrolyte for 2 h. Upon removal, residual electrolyte on the separator surface was gently blotted away with filter paper. The electrolyte uptake of the separator was then calculated according to Equation (2):

(2) $$E\mathrm{lectrolyte}\;\mathrm{uptake}\;\left(\%\right)\;=\;\frac{W\;-\;W_{0}}{W_{0}}\;\times \;100\%$$

where W is the mass of the wet separator after immersion in the electrolyte (g), and W_0_ is the mass of the dry separator (g).

Contact angle measurements were conducted using a JC2000D2M goniometer (Zhongyi Kexin Technology, Beijing, China), and the wettability of the separator surface was evaluated based on the measured contact angle values. The liquid electrolyte was slowly dropped onto the separator surface, contact angles were measured using the sessile drop method on a goniometer.

The separators were cut into circular disks with a diameter of 19 mm and subsequently heated in a vacuum oven at various predetermined temperatures for 0.5 h. The dimensional changes before and after thermal treatment were compared, and the thermal shrinkage rate of the separators was calculated according to Equation (3):

(3) $$\mathrm{Shrinkage}\;(\%)\;=\;\frac{S_{0}\;-\;S}{S_{0}}\;\times \;100\%$$

where S_0_ and S are the areas of the separator before and after heat treatment, respectively.

The separators were all cut into circular disks with a diameter of 19 mm. The masses of the three-layer composite separators and pristine PE separators were separately weighed. The areal mass loading of the coating was calculated according to Equation (4):

(4) $$\mathrm{Areal}\;\mathrm{mass}\;\mathrm{loading}\;=\;\frac{m_{\mathrm{coating}}\;-\;m}{A}$$

where m_coating_ (mg) and m (mg) are the mass of the three-layer composite separator and PE separator, respectively, and A (cm^2^) represents the area of the separator.

### 2.5. Electrochemical Measurements

The bulk impedance of symmetric stainless steel/separator/stainless steel coin cells was measured by electrochemical impedance spectroscopy (EIS). Spectra were recorded over a frequency range of 0.01 Hz to 10^6^ Hz with an AC amplitude of 5 mV. The ionic conductivity of the separator was subsequently calculated according to Equation (5):

(5) $$\sigma \;=\;\frac{L}{R_{b}\;\times \;A}$$

where σ (mS/cm) is the ionic conductivity, L (cm) is the thickness of the separator, R_b_ (Ω) is the bulk resistance obtained from electrochemical impedance spectroscopy, and A (cm^2^) is the surface area of the separator.

The impedance of LFP/separator/Li coin cells was measured by electrochemical impedance spectroscopy (EIS) over a frequency range of 0.01 Hz to 10^6^ Hz with an AC amplitude of 5 mV.

Cyclic Voltammetry (CV) was performed on LFP/separator/Li coin cells to investigate the electrochemical reactions of the electrode material during Li^+^ extraction and insertion. Measurements were carried out over a voltage range of 2.5–5.0 V at a scan rate of 0.1 mV/s.

LFP/separator/Li coin cells assembled with the various composite separators were allowed to rest in an argon-filled glove box for 24 h prior to electrochemical testing. Initial charge–discharge measurements were subsequently performed at a rate of 0.1 C using a LAND-CT2001C battery test system over a voltage window of 2.0–4.2 V.

Galvanostatic charge–discharge measurements are essential for assessing the cycle life, Coulombic efficiency, rate capability, and long-term cycling stability of LIB. In this work, charge–discharge cycling tests were conducted at a constant current rate of 2 C under ambient temperature for 400 cycles, and the cycling performance was evaluated over a voltage window of 2.0–4.2 V.

Rate capability refers to the discharge performance of a battery under varying current densities. During charge–discharge cycling, the specific capacity of LIBs typically declines as the applied current rate increases. The ability of a battery to retain a high specific capacity at elevated rates is therefore a critical metric for evaluating its electrochemical performance. In the rate capability measurements conducted herein, the cells were sequentially cycled at current rates of 0.2 C, 0.5 C, 1 C, 2 C, 3 C, 5 C, and finally 0.2 C.

### 2.6. Molecular Dynamics Simulation

In this study, the primary structural component of the composite separator consisted of blended nanofibers of PBI and PVDF-HFP. A commercial PE separator was used as the control sample, and a total of four simulation systems were established, namely 1PBIPH, 2PBIPH, 3PBIPH and pure PE separator systems. All separator models were set to a thickness of 10 Å. Each of the three PBIPH systems contained 3 PBI molecular chains and 3 PVDF-HFP molecular chains, while the PE system was constructed with 6 PE molecular chains. All molecular models were initially built using the Materials Studio (Version 23.1.0.3829) software package and subsequently imported into the LAMMPS platform for further simulation and computation [39].

Each simulation system was partitioned into three contiguous regions along the z-axis direction. The left region contained Li-free EC/DMC/EMC mixed electrolyte prepared at a volume ratio of 1:1:1; the central region corresponded to the 10 Å-thick separator; the right region was filled with the same EC/DMC/EMC electrolyte containing 10 lithium hexafluorophosphate (LiPF_6_)-derived Li^+^. Furthermore, an additional separator-free pure electrolyte system was constructed to calculate the Li desolvation energy. The separator was fixed at the center of the simulation cell to separate the bilateral electrolyte regions, which well mimicked the practical internal configuration of LIBs where the separator isolates the cathode and anode and enables Li migration.

All molecular dynamics simulations in this study were performed using the LAMMPS platform. The bonded and nonbonded interactions in the system were described by the Optimized Potentials for Liquid Simulations-All Atom (OPLS-AA) force field. The force field parameters for EC, DMC, and EMC were obtained from the classic general force field database [40], while those for Li^+^ were adopted from the work of Joung [41].

The whole simulation process consisted of two main stages: system equilibration and electric-field-driven ion transport. Firstly, structural optimization and system equilibration were implemented under the NPT ensemble at 298.15 K and 1 bar using the Nosé–Hoover thermostat and barostat. The equilibration process lasted for 2 ns to ensure that the density, total energy, and other key physical parameters of each system reached a stable and convergent state. During the simulation, carbon atoms at both ends of the separator molecular chains were fixed, while the remaining atoms were kept free to move. After pre-equilibration, the ensemble was switched to NVT, and a uniform electric field of 0.9 eV/Å was applied along the z-axis (perpendicular to the separator) to drive Li migration across the separator. The production simulation was conducted for 1 ns, and the final simulation data were statistically analyzed.

## 3. Results and Discussion

### 3.1. ^1^H NMR and FTIR Measurement

**Figure 4:**
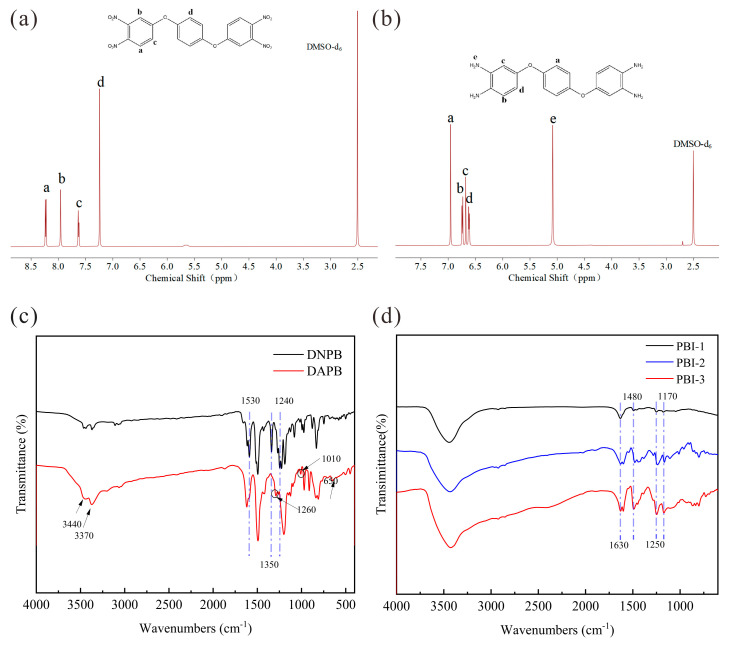
^1^H NMR spectra of (**a**) DNPB; (**b**) DAPB. FTIR spectra of: (**c**) DNPB and DAPB; (**d**) PBI.

To confirm the successful synthesis of the PBI materials, the synthesized products were characterized by ^1^H NMR and FTIR measurement. Figure 4a,b presents the ^1^H NMR spectra of DNPB and DAPB. The characteristic proton resonances of DNPB appear at 8.29 (ppm, d, 2H), 7.96 (ppm, s, 2H), 7.63 (ppm, d, 2H), and 7.24 (ppm, s, 4H). Following the nitro reduction process, the aromatic proton signals shift upfield, with the corresponding resonances observed at 6.95 (ppm, s, 4H), 6.74 (ppm, d, 2H), 6.67 (ppm, s, 2H), and 6.62 (ppm, d, 2H). Notably, a new signal emerges at 5.08 (ppm, s, 8H), which is assigned to the amino protons, thereby confirming the successful reduction in the nitro groups and the formation of DAPB [42].

Figure 4c presents the FTIR spectra of DNPB and DAPB. In the spectrum of DNPB, characteristic N=O stretching vibrations are observed at 1530 cm^−1^ and 1350 cm^−1^ [43], accompanied by an N–O stretching band at 1240 cm^−1^ [44]. The absorption bands located at 1260 cm^−1^ and 1010 cm^−1^ are attributed to C–O–C stretching modes [45], thereby corroborating the successful synthesis of DNPB. Upon conversion to DAPB, the N=O stretching bands at 1530 cm^−1^ and 1350 cm^−1^, as well as the N–O stretching band at 1240 cm^−1^, completely vanish. Concurrently, new stretching vibrations corresponding to –NH_2_ groups emerge at 3370 cm^−1^ and 3440 cm^−1^, together with an –NH_2_ bending vibration at 650 cm^−1^ [42]. These spectral changes unambiguously confirm the quantitative reduction of DNPB to DAPB.

Figure 4d presents the FTIR spectra of PBIs with varied structural architectures. Notably, the stretching vibration band of the carboxyl –C=O group at approximately 1700 cm^−1^ is completely absent [46], indicating full consumption of the dicarboxylic acid monomers. The absorption bands located at 1250 cm^−1^ and 1170 cm^−1^ are assigned to C–O–C stretching vibrations [47], whereas those at 1628 cm^−1^ and 1486 cm^−1^ correspond to –C=N– and –C–N stretching vibrations, respectively [48]. These spectral features collectively confirm the successful synthesis of PBIs possessing diverse molecular structures.

### 3.2. Solubility Measurement of PBIs

The solubility of the synthesized PBIs was evaluated in various organic solvents. Specifically, 2 g of NMP, DMF, DMAC, and DMSO were each placed into separate test tubes, and PBI powders were introduced at loadings of 5 wt% and 10 wt%. Solubility was assessed at 25 °C, and the corresponding results are summarized in Table 1. At ambient temperature, PBI-1 exhibited limited solubility in polar aprotic solvents—including NMP, DMF, DMAC, and DMSO—which is attributable to its rigid polymer backbone and extensive interchain hydrogen bonding. Complete dissolution of PBI-1 required elevated temperatures. In marked contrast, both PBI-2 and PBI-3 displayed substantially enhanced solubility in the same polar aprotic media, even at room temperature. This improved solubility originates from the increased content of ether functional groups along the polymer main chain, which imparts greater chain flexibility and disrupts the planar aromatic packing, thereby mitigating the tendency for chain aggregation [49].

### 3.3. Morphology and Areal Mass Loading of Separators

**Figure 5:**
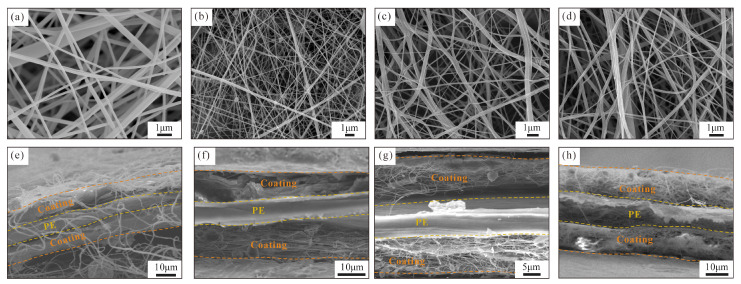
Microstructure of different composite separator fiber layers (**a**) PHPE; (**b**) 1PBIPHPE; (**c**) 2PBIPHPE; (**d**) 3PBIPHPE. Cross-section of the three-layer composite separator (**e**) PHPE; (**f**) 1PBIPHPE; (**g**) 2PBIPHPE; (**h**) 3PBIPHPE.

Figure 5a–d present SEM images of the electrospun composite fibrous layers fabricated from different materials. As evident from the micrographs, PBI-1, which possesses fewer ether functional groups within the polymer backbone, exhibits a relatively rigid molecular conformation and pronounced intramolecular hydrogen bonding. These characteristics confer poor processability, leading to discontinuous axial buckling during the electrospinning process. Consequently, localized morphological distortions arise, compromising the uniformity of the resulting fibrous layer. Inhomogeneities weaken the interfacial compatibility between the composite fiber layer and the underlying PE separator, reduce the overall structural uniformity of the separator, and ultimately have a negative impact on separator performance in LIBs. In marked contrast, as the content of ether functional groups increases, the electrospun fibers become more uniformly distributed and the fiber diameter tends toward a more consistent and stable value. Figure 5e–h presents SEM images of the cross-section of the three-layer composite separator. A distinct three-layer structure can be clearly observed in the cross-sectional morphology. The layers are tightly bonded without obvious gaps or delamination. The nanofiber layers uniformly coat both sides of the PE separator, and the interwoven fibers form abundant pore channels that provide sufficient pathways for Li^+^ transport. The intact composite structure demonstrates that the electrospinning technique enables effective combination of the functional layers and the substrate, and the as-prepared separator possesses excellent structural stability. The pristine PE separator has a thickness of 8 μm, while the as-fabricated three-layer composite separator reaches 27 μm in total. Accordingly, the total thickness of the fibrous layers on both sides is calculated to be 19 μm. The areal mass loading of the coating layers was measured, and the results are summarized in Table 2. It can be seen that the composite fibrous layers possess a relatively high areal mass loading with excellent uniformity.

### 3.4. Porosity and Wettability Measurements

**Figure 6:**
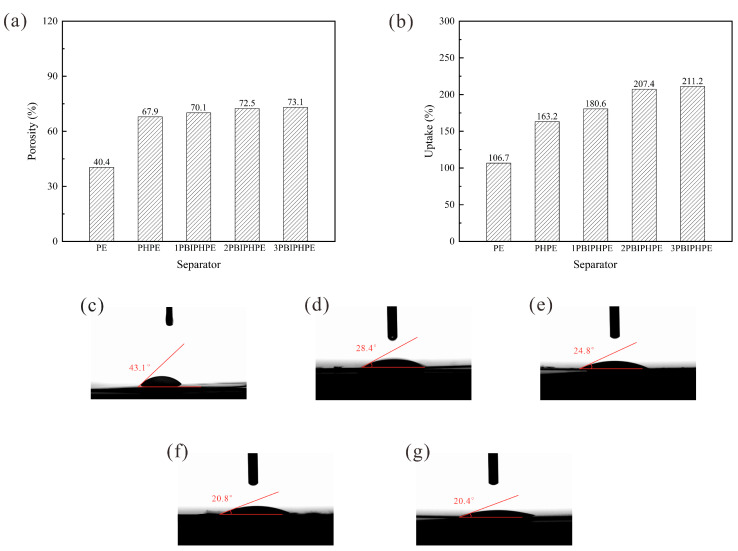
(**a**) Porosity of different separators; (**b**) electrolyte uptake of different separators. Contact angles of different separators (**c**) PE; (**d**) PHPE; (**e**) 1PBIPHPE; (**f**) 2PBIPHPE; (**g**) 3PBIPHPE.

The electrolyte is primarily accommodated within the porous architecture of the separator; consequently, the porosity exerts a profound influence on both electrolyte adsorption and wetting behavior. The porosity results are summarized in Figure 6a. The measured porosities of the PE, PHPE, 1PBIPHPE, 2PBIPHPE, and 3PBIPHPE separators are 40.4%, 67.9%, 70.1%, 72.5%, and 73.1%, respectively. The porosity of all three-layer separators is remarkably higher than that of the PE separator. This progressive enhancement in porosity is ascribed to the increasing content of ether functional groups within the PBI molecular structure. A higher density of ether groups promotes more uniform fiber distribution and stabilizes fiber diameter during electrospinning, thereby facilitating the formation of a more open and interconnected pore network.

Separators must possess excellent electrolyte wettability to facilitate rapid electrolyte infiltration into the porous structure and to establish uniform Li^+^ transport pathways. Such characteristics are essential for reducing internal resistance, enhancing charge–discharge kinetics, mitigating lithium dendrite proliferation, and improving the utilization efficiency of the active electrode materials [50]. As illustrated in Figure 6b, the electrolyte uptake values of the PBIPHPEs are 180.6%, 207.4%, and 211.2% for 1PBIPHPE, 2PBIPHPE, and 3PBIPHPE, respectively, all of which substantially exceed that of the pristine PHPE separator (163.2%) and PE separator (106.7%). The results of contact angle measurements are displayed in Figure 6c–g. The measured contact angles for the PHPE, 1PBIPHPE, 2PBIPHPE, and 3PBIPHPEs are 28.4°, 24.8°, 20.8°, and 20.4°, respectively, all values are higher than that of the PE separator (43.1°). The electrolyte uptake of PBIPHPEs increases gradually, while their contact angles decrease progressively. This phenomenon stems from the abundant ether functional groups, imidazole rings and nitrogen heteroatoms in the molecular structure of PBI. These structural moieties endow the polymer matrix with strong polarity, enabling excellent compatibility with polar electrolyte solvents. As a result, the interfacial tension between the separator surface and electrolyte is greatly reduced, and the PBIPHPEs exhibit remarkably improved electrolyte wettability compared with pristine PHPE separators.

### 3.5. Thermal Shrinkage of Separators

**Figure 7:**
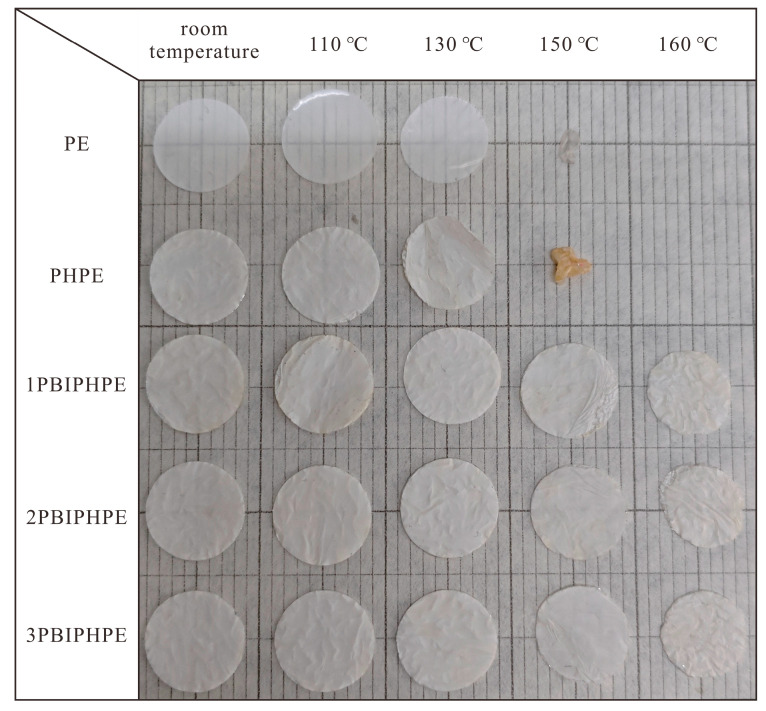
Photograph of separators after thermal treatment at various temperatures for 0.5 h.

During battery charge–discharge cycling, localized internal overheating may give rise to critical safety hazards. If the separator undergoes shrinkage, wrinkling, or even rupture upon thermal exposure, internal short circuits may directly ensue, and continued heat accumulation can ultimately trigger thermal runaway or catastrophic cell failure. Figure 7 summarizes the thermal shrinkage behavior of the various separators after isothermal treatment in an oven at designated temperatures for 0.5 h. At 110 °C, none of the four separator types exhibited discernible dimensional change. Upon elevating the temperature to 130 °C, the PHPE composite separator began to curl and deform, registering a shrinkage of 3%, PE separator exhibits a shrinkage of 8%. Further increasing the temperature to 150 °C resulted in complete failure of the PE and PHPE composite separator, whereas the three PBIPHPE composite separators displayed only marginal deformation. At 160 °C, all three PBIPHPEs manifested pronounced thermal shrinkage: 1PBIPHPE contracted by 18%, 2PBIPHPE by 20%, and 3PBIPHPE by 25%. This trend is rationalized by the increasing content of ether groups within the PBI repeating units. A higher density of ether linkages reduces the rigidity of the polymer backbone and promotes looser chain packing, which consequently leads to a modest increase in the observed thermal shrinkage ratio. These experimental findings unambiguously demonstrate that the PBI composite separators possess superior thermal stability, a characteristic that can substantially enhance the operational safety of LIBs.

### 3.6. Electrochemical Performance of Separators

**Figure 8:**
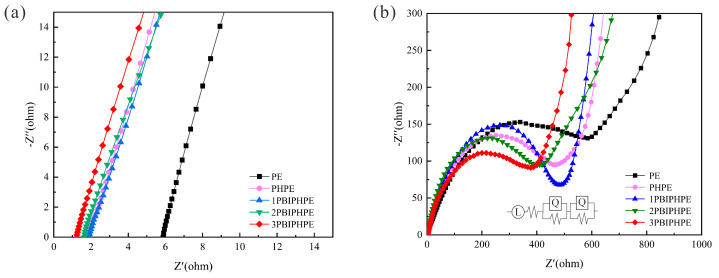
Electrochemical performance of batteries assembled with different separators: (**a**) bulk impedance spectrum (SS/separator-liquid electrolyte/SS cell); (**b**) alternating current impedance spectrum (LFP/Li cell). (L represents inductor, and Q denotes constant phase element (CPE).)

**Figure 9:**
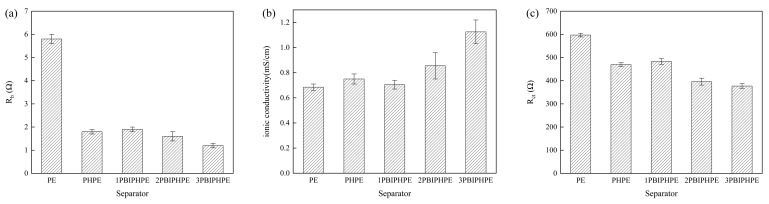
Electrochemical parameters of the separators: (**a**) bulk impedance (R_b_); (**b**) ionic conductivity (σ); (**c**) charge-transfer resistance (R_ct_).

Figure 8 and Figure 9 present the bulk impedance spectra, the corresponding ionic conductivity values and charge-transfer resistance of the various composite separators. All three-layer separators exhibit lower bulk impedance than the PE separator. Under the condition that all separators possessed comparable thicknesses (27 μm), the bulk impedance of the 1PBIPHPE three-layer composite separator was higher than that of the PHPE separator. In contrast, the bulk impedances of the other PBIPHPE composite separators were lower than that of PHPE and decreased progressively with increasing content of ether functional groups in the polymer backbone. Notably, all three-layer separators exhibit higher ionic conductivity than the PE separator. This behavior is rationalized as follows: the discontinuous axial buckling of PBI-1 fibers during electrospinning induced localized morphological distortions and diminished the uniformity of the fibrous layer. Such inhomogeneities compromised the interfacial compatibility between the composite fiber layer and the underlying PE separator, leading to elevated bulk impedance and a relatively low ionic conductivity of merely 0.706 mS/cm. Conversely, the 3PBIPHPE separator attained the highest ionic conductivity among the series, reaching 1.125 mS/cm.

To further assess the AC impedance characteristics, LFP/Li half-cells were assembled with the different separators and characterized by EIS using an electrochemical workstation. Figure 8b displays the Nyquist plots obtained for the various separators, which reflect the overall electrochemical impedance and Li^+^ diffusion behavior within the assembled LIBs. A smaller semicircle diameter along the Z′ axis is indicative of lower AC impedance. By comparing the semicircle diameters in the medium-frequency region, it is evident that only the 1PBIPHPE separator exhibited higher impedance than the PHPE counterpart. All other PBIPHPE composite separators displayed lower impedance values, which further diminished as the ether functional group content increased. Notably, the LIB assembled with the 3PBIPHPE composite separator exhibited an impedance of only 377 Ω. This marked improvement is attributed to the enhanced interfacial compatibility between the composite fibrous layer and the PE separator, which is facilitated by the greater abundance of ether functional groups in the PBI structure.

### 3.7. Electrochemical Performance of the Batteries

**Figure 10:**
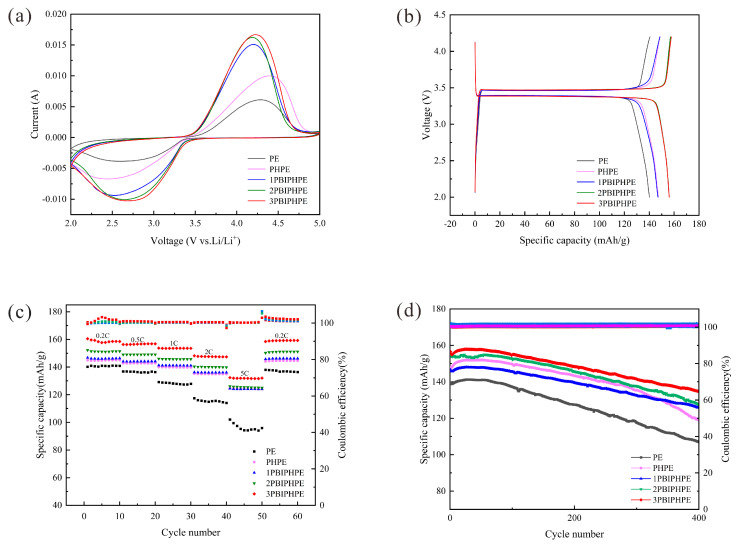
Electrochemical performance tests of LIBs assembled with different separators: (**a**) Cyclic Voltammetry (CV) curves; (**b**) first charge–discharge curves at 0.1 C rate; (**c**) rate capability test; (**d**) cycling performance at 2 C rate for 400 cycles.

CV measurements were conducted on LFP/Li coin cells to evaluate the electrochemical reversibility and polarization characteristics of the battery systems. At a given scan rate, a smaller potential separation between the anodic and cathodic peaks is indicative of enhanced reversibility and reduced polarization during the electrochemical reaction [51]. In CV measurements, the peak potential separation predominantly reflects the overpotential arising from electrolyte resistance, the presence of the separator impedes the migration of lithium ions between the cathode and anode, thereby elongating the ion conduction pathway and increasing the overall transport resistance [52]. To sustain the requisite ion flux and overcome this additional impedance, a supplementary driving force—manifested as an elevated applied potential—is required; this excess potential is formally defined as the overpotential. Consequently, the greater the ion transport resistance imposed by the separator, the more pronounced the peak potential separation observed in the CV profiles. Figure 10a presents the CV curves recorded for cells assembled with the different separators. Well-defined redox peaks are evident in all five profiles. Notably, the cell incorporating the PBIPHPE composite separator exhibits a substantially smaller peak potential separation than that equipped with the pristine PE and PHPE separator. This improvement is attributed to the polar active sites present within the PBI component, which interact synergistically with the polar functional groups of the solvent molecules. Such cooperative interactions effectively lower the desolvation energy barrier encountered during Li^+^ migration. As a result, the PBIPHPE-based cell displays a reduced peak potential separation relative to the PHPE-based reference. In particular, the cell assembled with the 3PBIPHPE composite separator yields the smallest peak potential separation, signifying that the 3PBIPHPE separator affords the optimal electrochemical reversibility and the lowest ion transport resistance. Furthermore, the redox peak currents observed for the cells containing PBIPHPE composite separators are markedly higher than those of the cell employing the PHPE separator, which reflects accelerated Li^+^ migration kinetics within the PBIPHPE systems. The absence of extraneous or impurity peaks across all four CV profiles confirms that each of the separators possesses excellent electrochemical inertness and does not participate in parasitic redox reactions, thereby ensuring stable and reliable cell operation.

Figure 10b presents the initial charge–discharge profiles of the assembled batteries recorded at a low rate of 0.1 C. The initial specific discharge capacities are 148.5, 148.7, 157.0, and 157.7 mAh/g for the PHPE, 1PBIPHPE, 2PBIPHPE, and 3PBIPHPEs, respectively, all of which are higher than that of the PE separator. All three PBIPHPEs deliver higher initial capacities than the PHPE control, with 3PBIPHPE achieving the highest value. This enhancement is attributed to the abundance of ether functional groups within the PBI-3 structure. The lone-pair electrons on the ether oxygen atoms can coordinate with Li^+^, promoting the dissociation of lithium salts and facilitating more efficient Li^+^ transport, which collectively contributes to the elevated specific capacity. This improved transport mechanism not only enhances the overall performance of the battery but also leads to greater cycle stability.

Figure 10c illustrates the rate capability of the half-cells. At a low rate of 0.2 C, the initial discharge capacities are 140.1, 145.0, 146.8, 151.2, and 159.5 mAh/g for the PE PHPE, 1PBIPHPE, 2PBIPHPE, and 3PBIPHPE separators, respectively. As the current rate is progressively increased to 5 C, the corresponding capacities correspond to capacity retention values of 70.7%, 83.9%, 84.8%, 84.1%, and 84.3%, respectively. The cell equipped with the 3PBIPHPE separator delivers the most favorable high-rate capability and capacity retention. This superior performance is primarily ascribed to the highest porosity and ionic conductivity exhibited by the 3PBIPHPE separator. Furthermore, the 3PBIPHPE structure incorporates a greater abundance of ether groups, which are capable of interacting with an increased number of Li^+^. These interactions facilitate the dissociation of the lithium salt and effectively reduce the migration resistance encountered by lithium ions, thereby contributing to the enhanced rate performance.

Figure 10d depicts the long-term cycling stability of the cells evaluated at a constant rate of 2 C over 400 cycles. After 400 cycles, the PHPE cell delivers a discharge capacity of only 119.2 mAh/g, corresponding to a capacity retention of 80.5%; the PE separator delivers inferior electrochemical performance (76.6%). In contrast, all PBIPHPE cells exhibit superior capacity retention: 1PBIPHPE retains 85.6%, 2PBIPHPE retains 82.7%, and 3PBIPHPE achieves the highest retention at 86.0%. The improved cycling stability of the PBIPHPEs is attributed to the intrinsically higher electrochemical stability of PBI relative to PVDF-HFP. Moreover, the highly uniform fibrous architecture of the 3PBIPHPE composite layer contributes to its optimal cycling performance.

### 3.8. Molecular Dynamics Simulation

**Figure 11:**
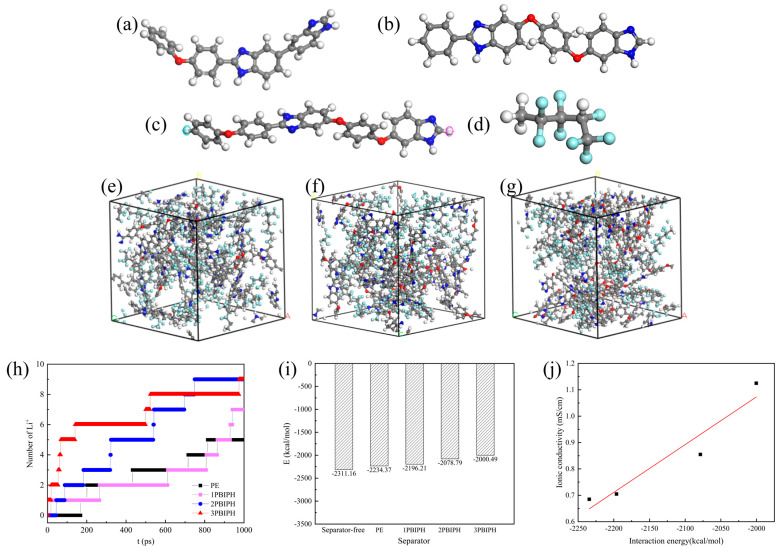
Structural model constructed via molecular dynamics simulations: (**a**) repeating unit of PBI-1; (**b**) repeating unit of PBI-2; (**c**) repeating unit of 3; (**d**) repeating unit of PVDF-HFP; (**e**) amorphous polymer unit cell system of 1PBIPH; (**f**) amorphous polymer unit cell system of 2PBIPH; (**g**) amorphous polymer unit cell system of 3PBIPH. (**h**) Number of Li^+^ penetrating different composite separators; (**i**) interaction energy between Li^+^ and electrolyte affected by separators; (**j**) linear fitting of interaction energy versus ionic conductivity.

In this study, molecular dynamics simulations were employed to investigate the influence of PBIPH composite separators on the transmembrane transport of lithium ions. Figure 11a–d displays the repeating units of PBI-1, PBI-2, PBI-3, and PVDF-HFP, respectively, while Figure 11e–g presents the amorphous polymer cell models constructed for the 1PBIPH, 2PBIPH, and 3PBIPH systems. To quantitatively characterize and compare the Li^+^ transport capabilities of the different separators, the number of lithium ions migrating from the right solvent reservoir across the separator into the left solvent reservoir was counted during a 1 ns electric-field-driven simulation. As illustrated in Figure 11h, the number of migrating Li^+^ recorded for the 2PBIPH separator system is substantially higher than that observed for 1PBIPH, and is comparable to the count achieved by the 3PBIPH system, all three separators exhibit better performance than the PE separator. The Li^+^ migration count refers to the total number of lithium ions that completely pass through the separator model and travel from one electrolyte reservoir to the other during the simulation; a higher count indicates more unobstructed ion transport pathways inside the separator, lower Li^+^ migration resistance and faster migration kinetics, which directly reflects the macroscopic ionic conductivity and rate capability of the separator [53]. The observed differences in Li^+^ transport performance among the separators are fundamentally governed by their distinct capacities to modulate the solvation environment confined within the pore network. In both the 2PBIPH and 3PBIPH systems, the density of ether groups and nitrogen-containing polar sites is sufficiently high to enable effective coordination with Li^+^ and to promote the dissociation of the lithium salt. Beyond this threshold, further increases in polar site density yield only marginal improvements in dissociation efficiency, resulting in similar ion migration counts for the two systems, albeit with a slight advantage conferred by 3PBIPH.

To further elucidate the intrinsic mechanism governing the differences in Li^+^ transport performance among the various separators from the perspective of molecular interactions, the adsorption energies of Li^+^ within the separator–solvent composite environment were calculated for the three separator systems, as presented in Figure 11i. The computed adsorption energies for the PE 1PBIPH, 2PBIPH, and 3PBIPH separators are −2234.37 kcal/mol, −2196.21 kcal/mol, −2078.79 kcal/mol, and −2000.49 kcal/mol, respectively. In the separator-free system, the value is −2311.16 kcal/mol. These values indicate that the binding strength of the separator–solvent composite system toward Li^+^ decreases progressively in the order PE > 1PBIPH > 2PBIPH > 3PBIPH, as reflected by the increasingly less negative adsorption energies. PBI, characterized by an abundance of benzene and imidazole rings along its backbone, exhibits outstanding thermal stability, chemical resilience, and mechanical robustness. Moreover, the electron-rich –C=N– can directly attract Li^+^ through favorable electrostatic interactions, thereby promoting the dissociation of the lithium salt, while the proton-donating –NH– are capable of forming hydrogen bonds with the solvent anions, effectively immobilizing them and consequently elevating the Li^+^ transference number. The lone-pair electrons resident on the –O– further engage in coordination with Li^+^, providing additional impetus for lithium salt dissociation. This robust interaction confers a dual benefit. On one hand, it stabilizes the solvation structure of Li^+^ and effectively reduces the desolvation energy barrier encountered during transmembrane migration. On the other hand, it constructs a continuous network of Li^+^ adsorption loci within the pore channels, thereby enabling efficient, low-barrier hopping transport of Li^+^ under the influence of an applied electric field [54]. The performance gradation observed among the three separators is also highly consistent with the adsorption energy trend. The 1PBIPH system affords only a moderate improvement in the solvation environment, yielding a basic enhancement in ion transport performance. In the 2PBIPH system, the solvent–Li^+^ adsorption is notably weaker, giving rise to a more continuous network of transport sites and correspondingly higher-level transport characteristics. The 3PBIPH system, distinguished by the least negative adsorption energy and thus the weakest solvent–Li^+^ binding interaction, not only maximizes the promotion of lithium salt dissociation and minimizes the migration energy barrier but also constructs the most continuous and efficient transport site network. Consequently, 3PBIPH delivers the optimal transmembrane Li^+^ transport capability.

Linear fitting between interaction energy and ionic conductivity was performed for PE, 1PBIPH, 2PBIPH and 3PBIPH, and the relevant results are presented in Figure 11j. The coefficient of determination R^2^ is calculated to be 0.92, demonstrating a strong linear correlation between the two parameters. This finding builds a reliable quantitative link between the microscopic interaction features and the macroscopic ionic conduction properties of the as-prepared separators.

## 4. Conclusions

A series of novel PBIs featuring ether functional groups within the repeating units were designed and synthesized. Three-layer composite separators with outstanding thermal stability and electrochemical performance were successfully fabricated by electrospinning PBI and PVDF-HFP composite layers onto both surfaces of PE separator. The incorporation of PBI significantly enhanced the thermal resilience and electrolyte wettability of the composite separators; notably, no discernible dimensional change was observed after thermal treatment at 150 °C for 0.5 h. Among the fabricated separators, the three-layer composite separator based on 3PBIPHPE delivered the optimal performance, characterized by a porosity of 73.1%, a high electrolyte uptake of 211.1%, an ionic conductivity of 1.078 mS/cm, and a specific charge capacity of 157.7 mAh/g. After 400 cycles at a 2 C rate, the capacity retention reached 86.0%, demonstrating electrochemical performance superior to that of the cell assembled with the PHPE three-layer separator. Molecular dynamics simulations were conducted to establish a structural model of the composite separator. During the 1 ns simulation under an applied electric field of 0.9 eV/Å, the number of Li^+^ migration events recorded for the PBIPH separator system was higher than that of PE. The adsorption energies of Li^+^ within the separator–solvent composite environment were calculated for the three systems, revealing that the solvent binding strength toward Li^+^ decreases progressively in the order 1PBIPH > 2PBIPH > 3PBIPH. Overall, the 3PBIPH separator system exhibited the best comprehensive performance, with only a marginal performance gap relative to the 2PBIPH system. This is because the amounts of ether groups and nitrogen-containing polar sites in 2PBIPH and 3PBIPH are sufficiently high to efficiently coordinate with Li^+^ and promote the dissociation of lithium salts. As a result, the improvement in dissociation efficiency is no longer significant, and the adsorption interaction is not remarkably enhanced. The molecular dynamics simulation results were in excellent agreement with the experimental findings obtained in this study. Consequently, the newly developed PBIPH composite separator exhibits outstanding performance in LIBs, effectively enhancing both the operational safety and the electrochemical properties of the devices.

## Figures and Tables

**Table 1:** Solubility of different PBIs.

	NMP	DMF	DMAC	DMSO
PBI-1	+	+	+	+
PBI-2	++	++	++	++
PBI-3	++	++	++	++

“+” indicates solubility at 5 wt%; “++” indicates solubility at 10 wt%.

**Table 2:** Areal mass loading of the composite separator.

	A (cm^2^)	m (mg)	Areal Mass Loading (mg/cm^2^)
PE	2.0096	1.2 ± 0.1	-
PHPE	2.0096	2.5 ± 0.2	0.646 ± 0.15
1PBIPHPE	2.0096	2.5 ± 0.3	0.647 ± 0.19
2PBIPHPE	2.0096	2.7 ± 0.2	0.747 ± 0.15
3PBIPHPE	2.0096	2.7 ± 0.2	0.747 ± 0.15

## Data Availability

The original contributions presented in this study are included in the article. Further inquiries can be directed to the corresponding author.
